# Detection of *K-ras* Mutations in Predicting Efficacy of Epidermal Growth Factor Receptor Tyrosine Kinase (EGFR-TK) Inhibitor in Patients with Metastatic Colorectal Cancer

**DOI:** 10.1371/journal.pone.0101019

**Published:** 2015-05-07

**Authors:** Ze Li, Xue-Wei Liu, Zhao-Cheng Chi, Bao-Sheng Sun, Ying Cheng, Long-Wei Cheng

**Affiliations:** Department of Gastrointestinal Surgery, Cancer Hospital of Jilin Province, Changchun, Jilin, China; Hungarian Academy of Sciences, HUNGARY

## Abstract

Epidermal growth factor receptor tyrosine kinase (EGFR-TK) inhibitors are useful in treating different advanced human cancers; however, their clinical efficacy varies. This study detected *K-ras* mutations to predict the efficacy of EGFR-TK inhibitor cetuximab treatment on Chinese patients with metastatic colorectal cancer (mCRC). A total of 87 patients with metastatic colorectal cancer were treated with cetuximab for 2-16 months, in combination with chemotherapy between August 2008 and July 2012, and tissue samples were used to detect *K-ras* mutations. The data showed that *K-ras* mutation occurred in 27/87 (31%). The objective response rates and disease control rate in *K-ras* wild type and mutant patients were 42% (25/60) versus 11% (3/27) (p<0.05) and 60% (36/60) versus 26% (7/27) (p<0.05), respectively. Patients with the wild-type *K-ras* had significantly higher median survival times and progression-free survival, than patients with mutated *K-ras* (21 months versus 17 months, p=0.017; 10 months versus 6 months, p=0.6). These findings suggest that a high frequency of *K-ras* mutations occurs in Chinese mCRC patients and that *K-ras* mutation is required to select patients for eligibility for cetuximab therapy. Further prospective studies using a large sample size are needed to confirm these preliminary findings.

## Introduction

Colorectal cancer (CRC) is the third most commonly diagnosed cancer in the world and is one of the most significant health problems in China [1]. Although the incidence of CRC used to be lower in China than in Western countries, it has increased rapidly in recent years [2]. Surgery is the best treatment option for CRC, like most other cancers, but metastatic CRC needs combination therapy, such as surgery plus chemotherapy or target therapy. During the past decades, 5-fluorouracil (5-Fu) regimens have produced median survival of approximately 12 months for advanced CRC, while calcium folinate (CF) plus 5-Fu prolongs median survival to 14 months [3]. Furthermore, oxaliplatin and irinotecan have increased the median overall survival of patients to more than 20 months [4]. Most recently, target therapy, including anti-epidermal growth factor receptor tyrosine kinase (EGFR-TK) has been shown to improve overall survival of patients with wild-type KRAS (v-Ki-ras2 Kirsten rat sarcoma viral oncogene homologue) metastatic CRC [5]. However, anti-EGFR-TK therapy using gefitinib, erlotinib, or cetuximab produces different results in different human cancers. The reason may be because anti-EGFR-therapy in patients with mutated *K-ras* may not only be ineffective but also detrimental [5]. Thus, 2011 guidelines from the National Comprehensive Cancer Network (NCCN) have recommended cetuximab as first-line therapy for patients with the wild-type *K-ras* since EGFR and *K-ras* mutations are exclusive [6].

*K-ras* gene encodes a 21 kDa protein, which is a GTP/GDP binding protein with GTPase activity and is involved in transduction of mitogenic signals to link receptor tyrosine kinase activation to downstream effectors. After GDP binds to the p21 RAS protein, it will convert it into an inactive form, losing its function for signal transduction. Mutations of the RAS gene usually cause constitutive activation of RAS GTPase, leading to activation of the downstream signaling pathways and resulting in cell transformation and tumorigenesis [7–9]. In CRC, more than 90% of *K-ras* mutations occur in *K-ras* exon 1 codon 12 and codon 13 [7,8].

Cetuximab is a chimeric mouse/human monoclonal antibody against EGFR-TK and the development and use of cetuximab have improved survival of mCRC patients. Previous data indicated that the effect of cetuximab was tightly associated with *K-ras* mutations, so the US Food and Drug Administration recommended that patients should undergo *K-ras* mutation analysis before receiving cetuximab treatment. However, not all patients with wild-type *K-ras* will benefit from cetuximab treatment, as there was no association between EGFR expression and cetuximab efficacy. The overall response rate of patients with wild-type *K-ras* to cetuximab is only 40–60%, but the response rate of patients with *K-ras* mutations was only 10% or less [10,11], thus, in this study, we detected *K-ras* mutations to predict the efficacy of EGFR-TK inhibitor cetuximab in Chinese patients with metastatic colorectal cancer.

## Materials and Methods

### Patients

In this study, we recruited a total of 87 patients with histologically confirmed mCRC in Jilin Provincial Cancer Hospital between January 2008 and August 2010 who were treated with weekly cetuximab (400 mg/m^2^ as an initial loading dose, and 250mg/m^2^ subsequent dose) in combination with chemotherapy (standard dose). Specifically, 55 patients received cetuximab plus oxaliplatin-based chemotherapy and an additional 32 patients received cetuximab plus irinotecan-based chemotherapy for 2–16 months. Cetuximab was administered as first-line treatment in all 87 patients weekly until disease progression or the end of this study. The Cancer Hospital of Jilin Province review board approved this study and written informed consents were obtained from all the subjects. However, patients were excluded from this study if they had not received postoperative chemotherapy, or if they were < 25 or > 80 years old.

### Evaluation of treatment response and survival of patients

Treatment response was estimated every two months by computed tomography (CT) of the site of the metastasis (the liver and lung) according to the Response Evaluation Criteria in Solid Tumors (RECIST) [12]. Patients were categorized as a complete response (CR), partial response (PR), stable disease (SD), or progressive disease (PD). The objective response rate was defined as (CR+PR)/Total, while the disease control rate was defined as (CR+PR+SD)/Total. Overall survival was defined as the time from histopathological diagnosis to death from any cause. The stable disease indicted that after treatment, the disease would persist for at least four weeks without significant change in tumor lesion. The last follow up of these patients was in July 2012. However, all subjects in this study had stage IV disease and if the disease progressed, the treatment with cetuximab would be stopped. Thus, the study presents progression-free survival data.

### DNA extraction and detection of K-ras mutations

Paraffin blocks from tumor tissues were re-assessed using H&E-stained tissue sections to ensure that the total number of tumor cells was similar for different samples. Selected tissue sections from CRC patients containing a high proportion of tumor cells (typically ≥ 60%) were identified by three experienced pathologists. After that, genomic DNA was then extracted from three 10 μm-thick sections of each tumor sample according to protocols described previously [13]. Briefly, the tissue sections were deparaffinized in xylene, washed in absolute ethanol, and incubated at room temperature with 150 μl of digestion buffer (1 mg/ml proteinase K; 0.05 M Tris-HCl, pH 8.5; 1 mM ethylenediaminetetra-acetic acid; 0.5% Tween 20; 2% sodium dodecyl sulphate) for 24 h. The next day, the reactions were centrifuged at 15 000 g for 15 min and DNA was extracted by using a QIAamp DNA FFPE Tissue Kit (Qiagen GmbH, Hilden, Germany) according to the manufacturer’s instructions. After that, the DNA concentration was spectrophotometrically assessed using a wavelength of 280/260 nm and adjusted to a final concentration of 100 μg/ml and stored at −20°C until use.

To detect *K-ras* mutations, polymerase chain reaction-single strand conformation polymorphism (PCR-SSCP) was performed using primers and a PCR kit (Takara Biotechnology (Dalian) Co. Ltd, Dalian, China). Briefly, a 50-μl PCR mixture was prepared for each sample, which contained 2 μl genomic DNA, 1 μl of 10 μmol/l of each primer and 25 μl premix (1.5 U Takara Taq polymerase, 10x PCR buffer, 3.0 mmol/l Mg2+, 0.4 mmol/l deoxynucleotidetriphosphate). The primers to detect *K-ras* mutations at codons 12 and 13 were 5'-AGGCCTGCTGAAAATGACTGAATA-3' and 5'-CTGTATCAAAGAATGGTCCTGCAC-3', as described previously [14]. PCR was performed on a 7900HT Fast Real-Time PCR System (Applied Biosystems, Foster City, CA, USA) with an initial denaturing step at 94°C for 5 min, followed by 30 cycles of 94°C for 30 sec, 60°C for 30 sec, and 72°C for 30 sec, a final 72°C extension for 5 min, and then stored at 4°C indefinitely. After that, the PCR products were separated on 1.5% agarose gels and purified using a QIAquick PCR Purification Kit (Qiagen, Venlo, The Netherlands) and sent for DNA sequencing analysis of the *K-ras* mutations at the Shanghai Generay Biotech Co. Ltd. (Shanghai, China).

### Statistical analysis

The association between *K-ras* mutations and the response to cetuximab treatment was analyzed by using Fisher’s exact test. Kaplan-Meier curves and the Log-rank test were used to analyze the association between *K-ras* mutations and the overall survival (OS) and progression-free survival (PFS) of patients. The Cox proportional hazard model was utilized to determine the factors related to overall survival. Statistical analysis was performed by using SPSS 17.0 (SPSS Inc., Chicago, IL, USA). P<0.05 was considered as statistically significant.

## Results

In this study, we treated a total of 87 mCRC patients with cetuximab plus oxaliplatin-based (55 patients) or irinotecan-based (32 patients) chemotherapy for 2 to 16 months. The tumor tissues were subjected to detection of *K-ras* mutations. We found 27 patients with a mutated *K-ras* and the overall *K-ras* mutation rate was 31%. The detailed patient characteristics are shown in Table 1.

**Table 1:** Clinicopathological characteristics of patients with metastatic colorectal cancer.

Clinicopathological characteristics	n = 87	%
Gender		
Male	57	65.5
Female	30	34.5
Age (yrs.) 63 (28–86)		
≤ 63	42	48.3
> 63	45	51.7
Growth pattern		
Polypoid	26	29.9
Ulcer	61	70.1
Tumor location		
Colon	58	66.7
Rectum	29	33.3
Tumor differentiation		
Poor*	30	34.5
Well	57	65.5
Sites of metastatic CRC		
liver	65	74.7
lung	12	13.8
others**	10	11.5
Combined chemotherapy		
Oxaliplatin-based	55	63.2
Irinotecan-based	32	36.8
*K-ras* status		
Wild	60	69
Mutation	27	31

*Poor: including poorly differentiated adenocarcinoma, signet ring cell carcinoma and mucinous adenocarcinoma;

**Including ovary, subcutaneous of abdominal wall, pelvic cavity.

The patients with wild-type *K-ras tumor* had better ORR and DCR than that of patients with mutated *K-ras tumor*, i.e., DCR, 60% vs. 26% (χ^2^ = 23.582, p<0.05), ORR, 42% vs. 11% (χ^2^ = 24.669, p<0.05, Table 2), indicating that detection of *K-ras* mutation can predict the response of the patients to cetuximab plus chemotherapy. Among 32 cases receiving irinotecan, nine cases had *K-ras* mutation, while among 55 cases receiving oxaliplatin, 18 cases had *K-ras* mutation; however, there was no significant difference of *K-ras* mutation between patients receiving irinotecan and those receiving oxaliplatin (p>0.05, Table 3). Moreover, the patients treated with cetuximab plus irinotecan-based chemotherapy had better ORR and DCR than that of patients treated with cetuximab plus oxaliplatin-based chemotherapy, i.e., DCR, 66% vs. 40% (χ^2^ = 13.569, p<0.05), ORR, 38% vs. 29% (χ2 = 1.818, p>0.05, Table 3).

**Table 2:** *K-ras* mutation prediction of the clinical response of patients after treatment with Cetuximab plus chemotherapy.

*K-ras*	CR	PR	SD	PD	ORR	χ^2^	*p* value	DCR	χ^2^	*p* value
WT (60)	10	15	11	24	42%	24.669	<0.05	60%	23.582	<0.05
MT (27)	0	3	4	20	11%			26%		

**Table 3:** ORR and DCR in patients treated with Cetuximab plus irinotecan-based chemotherapy and Cetuximab plus oxaliplatin-based chemotherapy.

	CR	PR	SD	PD	ORR	χ^2^	*P* value	DCR	χ^2^	*P* value
Oxaliplatin-based (55)	4	12	6	33	29%	1.818	>0.05	40%	13.569	<0.05
Irinotecan-based (32)	6	6	9	11	38%			66%		

**Fig 1:**
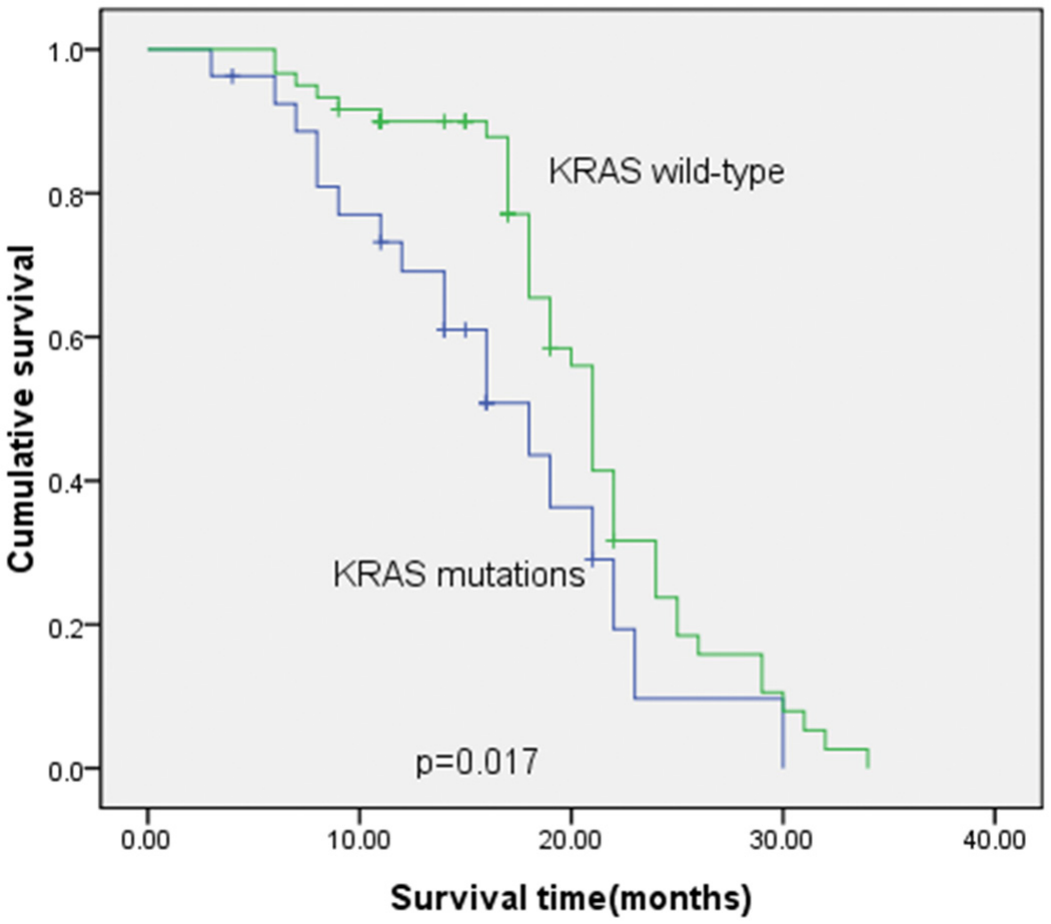
Kaplan-Meier survival curves of patients with wild type (n = 50) vs. mutant (n = 27) *K-ras*.

Furthermore, we found that the median survival time was 21 months in patients with wild-type *K-ras*, whereas the median survival time was 17 months in patients with mutated *K-ras*. This difference was statistically significant (χ^2^ = 5.703, P = 0.017; Fig 1). The progression-free survival was 10 months in patients with wild-type *K-ras*, whereas the progression-free survival was 6 months in patients with mutated *K-ras*. This difference was not statistically significant.

**Fig 2:**
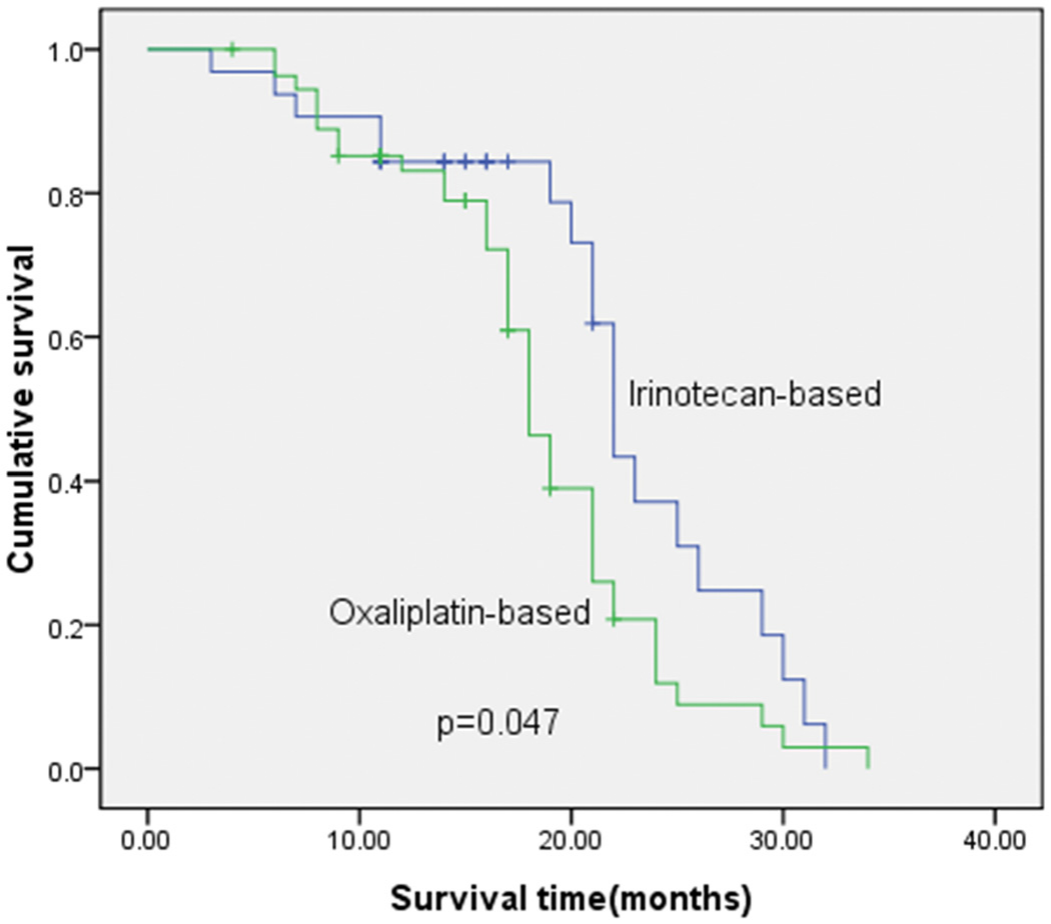
Kaplan-Meier survival curve analysis of treatment efficacy with survival of 87 mCRC patients. These patients were treated with irinotecan-based (n = 32) and oxaliplatin-based (n = 55).

**Fig 3:**
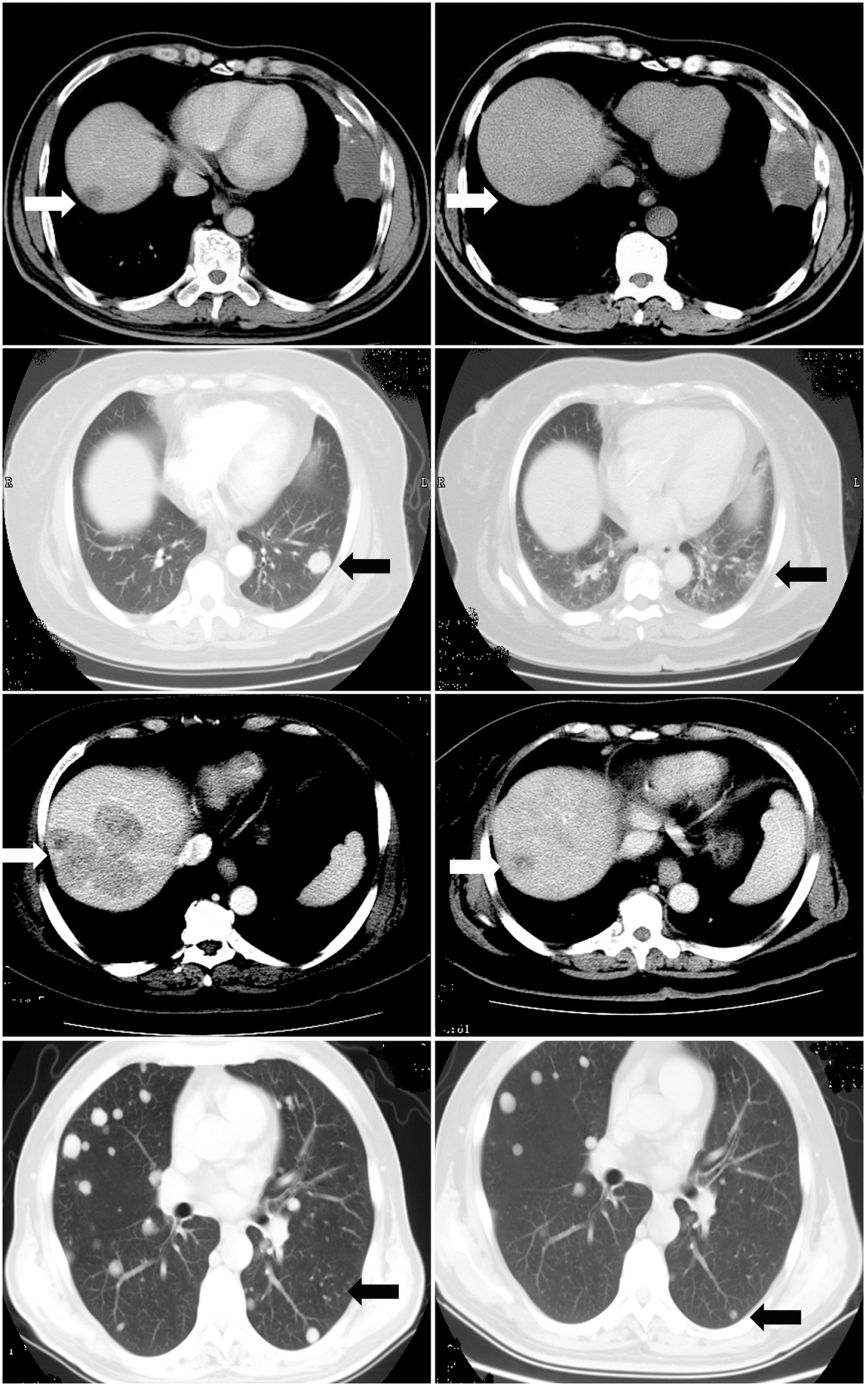
CT imaging of mCRC patients before and after treatment. Case 1 is a CR patient who was treated with four cycles of cetuximab plus irinotecan-based chemotherapy. Case 2 is a CR patient who was treated with three cycles of cetuximab plus oxaliplatin-based chemotherapy. Case 3 is a PR patient who received four cycles of cetuximab plus irinotecan-based chemotherapy. Case 4 is also a PR patient who was treated with four cycles of cetuximab plus oxaliplatin-based chemotherapy. CR, completely response; PR, partial response.

Moreover, the median survival time was 22 months in patients treated with cetuximab plus irinotecan-based chemotherapy, while the median survival time was 18 months in patients treated with cetuximab plus oxaliplatin-based chemotherapy This difference was also statistically significant (χ^2^ = 3.957 p = 0.047; Fig 2). After CT and nuclear magnetic resonance imaging, the mCRC patients received cetuximab plus irinotecan or cetuximab plus oxaliplatin chemotherapy and four mCRC patients were shown in Fig 3. Specifically, in Case 1, the CR (completely response) patient was treated with four cycles of cetuximab plus irinotecan-based chemotherapy. Case 2 was a CR patient who was treated with three cycles of cetuximab plus oxaliplatin-based chemotherapy. Case 3 was a PR (partial response) patient who received four cycles of cetuximab plus irinotecan-based chemotherapy. Case 4 was also a PR patient who was treated with four cycles of cetuximab plus oxaliplatin-based chemotherapy. The liver focus disappeared in Case 1 and 2, and the focus was greatly decreased in Case 3 and 4. The univariate analysis showed that *K-ras* mutations and treatment choice were associated with poor prognosis in CRC patients (*p* = 0.017 and, *p* = 0.047, respectively), while the multivariate analysis showed that, *K-ras* mutation, treatment choice, and poor tumor differentiation were independent factors for survival of patients with mCRC (*p* = 0.004, *p* = 0.006 and *p* = 0.015, respectively; Table 4).

**Table 4:** Multivariate Cox proportion hazard model analysis of clinicopathological factor association with prognosis of 87 mCRC patients.

Characteristic	RR	95% CI	p value
Age	0.849	0.473–1.524	0.583
Sex	0.261	0.643–2.126	0.609
*K-ras*	0.390	0.207–0.735	0.004
Location of tumor	1.174	0.463–2.974	0.735
Growth pattern	1.220	0.635–2.343	0.551
Differentiation	0.413	0.015–0.413	0.015
Combined chemotherapy	2.826	1.356–5.892	0.006
Sites of metastatic	1.494	0.551–4.054	0.430

## Discussion

In the current study, we detected *K-ras* mutations in tumor tissues to predict the response of Chinese patients with metastatic colorectal cancer to cetuximab plus oxaliplatin- or irinotecan-based chemotherapy. We found that patients with wild-type *K-ras* after these treatments had better ORR and DCR than patients with mutated *K-ras* (42% vs. 11% and 60% vs. 26%, respectively.) Moreover, we found that the median survival time of patients with wild-type *K-ras* was 21 months compared to 17 months in patients with mutated *K-ras* (p = 0.017). In addition, the median survival time of patients treated with Cetuximab plus irinotecan-based chemotherapy was 22 months compared to 18 months in patients treated with cetuximab plus oxaliplatin-based chemotherapy (p = 0.047). This study supports the observation that detection of *K-ras* mutation is a useful predictor for anti-EGFR-TK treatment in metastatic colorectal cancer. Further study should investigate the underlying molecular mechanism responsible for mutated *K-ras* in the progression of advanced colorectal cancer.

Colorectal cancer frequently metastasizes to the liver, which leads to difficulty in controlling it clinically. In our study, there were 65 of 87 patients (74.7%) who had metastatic disease in the liver, followed by metastasis to the lung, ovary, and skin. Without progressive treatment, patients with metastatic disease have a very poor prognosis [15]; thus, advances in treatment of metastatic colorectal cancer will save lives and improve the quality of life for patients. In this study, we assess *K-ras* mutations in 87 mCRC patients to predict the response of patients to cetuximab plus oxaliplatin- or irinotecan-based chemotherapy. We found 27 patients with mutated *K-ras* (31%), which was consistent with those in western CRC patients [16] and Chinese CRC patients [17]. Previous studies have demonstrated that patients with wild-type *K-ras* had a better response to cetuximab treatment than those with mutated *K-ras* [10,11]. In the current study, we also confirm these findings [10,11,18]. At a molecular level, other non-tyrosine kinases may also activate the EGFR signaling pathway that might explain why *K-ras* mutation is useful, but not perfect in predicting resistance to cetuximab. Moreover, we, or others, just detected the most common mutations in *K-ras* (i.e., exon codon 12 and 13), but other *K-ras* may also play a role in anti-EGFR-TK resistance. In addition, it is necessary to verify whether the subtypes or site of metastatic colorectal cancer play a role in response to anti-EGFR-TK therapy, like cetuximab.

Furthermore, the current study combined cetuximab with oxaliplatin- or irinotecan-based chemotherapy, which may also affect the response of the patients. The difference we show in this study may be due to the drug interaction between cetuximab and irinotecan or oxaliplatin; thus, further study is needed to clarify this issue. However, Van Cutsem et al. investigated chemotherapy outcome in K-*ras* patients with metastatic colorectal cancer and showed that there was no significant difference in progression-free survival or overall survival between patients who received cetuximab plus irinotecan, fluorouracil, and leucovorin (FOLFIRI) or FOLFIRI alone [19]. Therefore, it is possible that a different chemotherapy regimen may lead to different responses in patients.

Activated K-ras protein interacts with phosphatidylinositol-3 kinase (PI3K) to activate its downstream effectors (such as mammalian target of rapamycin) to indirectly modulate cell survival. Moreover, through the Braf/mitogen-activated kinase (MAPK) pathway, activated K-ras protein also influences cell proliferation [20]. To date, a number of targeted therapies against EGFR signaling have been established and cetuximab is a human/mouse chimeric IgG1 monoclonal antibody that binds to the extracellular domain of the EGFR and inhibits EGFR-mediated signaling [21]. A recent study showed that cetuximab treatment was effective in wild type *K-ras* and EGFR Chinese CRC patients, which provided evidence for efficacy-prediction of EGFR targeting therapeutic strategies [22,23].

However, much more is needed for successful cetuximab therapy of advanced CRC patients in the clinic. We could first assess *K-ras* mutations to select patients eligible for cetuximab treatment and then investigate how mutated *K-ras* antagonizes cetuximab efficacy or bypasses EGFR-TK inhibition. A novel drug combination may be needed to suppress activity of mutated K-ras protein.
